# The Utilization of Linear Polylysine Coupled with Mechanic Forces to Extract Microbial DNA from Different Matrices

**DOI:** 10.3390/microorganisms8121901

**Published:** 2020-11-30

**Authors:** Celia François, Celia Martinez, Clement Faye, Nathalie Pansu, Catherine Dunyach-Remy, Laurent Garrelly, Benoit Roig, Axelle Cadiere

**Affiliations:** 1 Gl-Biocontrol, 34830 Clapiers, France; c.francois@gl-biocontrol.com (C.F.); c.martinez@gl-biocontrol.com (C.M.); c.faye@gl-biocontrol.com (C.F.); l.garrelly@gl-biocontrol.com (L.G.); 2 Institute National de la Santé et de la Recherche Médicale, U1047, University Montpellier, UFR de Médecine, 30908 Nimes, France; n-pansu@chu-montpellier.fr (N.P.); catherine.remy@chu-nimes.fr (C.D.-R.); 3 Department of Microbiology, CHU Nimes, University Montpellier, 30029 Nimes, France; 4 EA7352 CHROME, University Nimes, Rue du Dr G. Salan, CEDEX 1, 30021 Nimes, France; benoit.roig@unimes.fr

**Keywords:** linear polylysine, purification DNA, PCR inhibitors, complex samples

## Abstract

Molecular approaches are powerful tools that are used for medical or environmental diagnoses. However, the main limitations of such a tools are that they extract low levels of DNA and they do not remove the inhibitors of polymerase chain reaction (PCR). Although the use of polycation to complex and purify DNA has been described in the literature, elution often requires a high ionic strength or pH levels not compatible with molecular analyses. In this paper, we described a new process that is based on the complexation of DNA with linear polylysine, followed by capturing the complex by a cation exchange resin. The originality of the process consisted of using mechanic force to elute DNA from the complex. The extraction method showed several advantages when compared to existing methods, such as being compatible with pH levels that range from 5 to 11, as well as high levels of DNA recovery and elimination of PCR inhibitors from complex samples. This method was successfully applied to different types of samples, such as environmental samples, beverage samples, and medical samples. Furthermore, it was proven to be a good solution for removing PCR inhibitors and assuring good DNA recovery yield.

## 1. Introduction

Molecular detection methods, such as qPCR (quantitative polymerase chain reaction), are powerful and promising diagnostic tools in the medical and environmental fields. However, due to the complexity of samples, extracting and purifying nucleic acid is often the limiting factor in these methods [1,2,3]. The presence of inhibitory elements in samples that are not eliminated is one of the major drawbacks. A large range of PCR inhibitors have been reported in the literature, in particular, organic compounds, such as urea and humic acid [4,5]. 

Several methods have been described in order to remove inhibitors. One particularly effective method was based on serially diluting a sample [6,7]. However, this solution dilutes the target of interest, which results in sensitivity loss and a limit of detection increase. Consequently, it is not adapted for low concentration samples.

Other methods of extracting and purifying DNA have been previously described in the literature, such as the use of phenol–chloroform–isoamyl alcohol (25:24:1), as well as chromatography with Sephadex G100 or G200 columns. These techniques have been used in order to remove salts and small proteins [8,9]. The main limitations concern low efficiency and variable extraction yields according to the matrix composition [10,11]. In addition, these approaches are time consuming and not easy to perform. In addition, the use of several solvents (such as phenol and chloroform) can make these approaches unsafe, while supplementary steps are required for removing inhibitors that increase the extraction time [12].

During the last decade, solid phase extraction has become the most widespread method in laboratories, in particular the spin column with silica membrane or magnetic beads. These methods allow for one to obtain good DNA purity [13], but interfering molecules such as guanidine hydrochloride or alcohol could inhibit PCR. Moreover, these techniques showed different performances in terms of recovery efficiency.

Polycations are also largely described in the literature in order to interact with DNA and they were principally applied to studies regarding DNA delivery [14] and DNA purification. Of note, DNA elution from complex DNA–polycation [15,16] was the main drawback in this last case.

The variation of ionic strength is a good option for obtaining purified DNA; however, the presence of high salt concentrations in the elution buffer is not recommended for molecular techniques, such as PCR.

Other elution options that have been tested are the modifications of complex charges by varying the pH. In this approach, DNA is captured on a pH that is below the polycation pKa and released with a higher pH [17]. The requirement of two pH impeded the development of this method, because the pH of an elution buffer was incompatible with the PCR procedure. More recently, Pandit et al. [18,19] demonstrated that chitosan-based microparticles could be used at pH 8.5 for both capturing and elution. 

**Figure 1:**
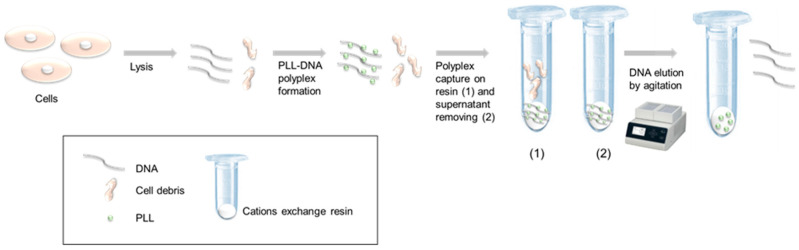
Principe of DNA extraction and purification while using DNA-polylysine (PLL) resin complex. (1) polyplex was captured on resin (2) supernatant was removed.

In this work, we used linear polylysine (PLL) as a polycation in order to capture and purify DNA. Our process was based on the (1) formation of a DNA-PLL complex, followed by (2) fixating the complex on cation exchange resin, and, finally, on (3) DNA elution. For the first time, we demonstrated that DNA elution from the complex was possible while only using mechanical force (vortex) (Figure 1). Moreover, we verified that the pH of DNA capture did not impact the capture itself nor the yield of DNA recovery. 

In the first approach, we defined the best parameters to realize the extraction and assessed whether the process was sensitive to some parameters (e.g., amount of DNA, pH of sample, salt concentration, etc.). Subsequently, we assessed the performance of the protocol by applying it to cooling tower samples. In these samples, the presence of inhibitors was known for its interferences and it represented a limit in qPCR detection [20]. In order to evaluate the efficiency of our method, we referred to the normative XPT 90-471 (April 2006) that fixes the performances of DNA extraction process for this kind of samples. Given the results that were obtained on the cooling towers samples, we hypothesized that the protocol could be applied to matrices that pose problems for microbiota or environmental DNA extraction. Therefore, we proposed some adaptations of the process that could be applied to DNA extraction in such matrices.

This new methodology was able to easily eliminate most PCR inhibitors in different samples and it had great potential on DNA extraction from complex matrices, e.g., bacterial microbiota or environmental DNA studies. Contrary to existing commercial kits that were specific to one matrix (soil, feces, blood, or water), we showed that small adaptations of this protocol allowed for the extraction of DNA with a high yield and that is devoid of PCR inhibitors from different matrices. 

## 2. Materials and Methods 

### 2.1. Linear Polylysine (PLL) Synthesis

PLL was synthetized by COLCOM (Clapiers, France) via the polycondensation of Lysine-N-Carboxyanhydrides. The counter ion was a trifluoroacetic acid (TFA). This PLL had a degree of polymerization that ranged between 80 and 120 amino acids, featuring an average molecular weight of 28,320 Daltons. The polydispersity was estimated at 1.072 while using an exclusion size chromatography that was coupled with a multi-angle laser light.

### 2.2. Bacterial Culture and DNA Extraction

*Legionella pneumophila* serogroup 1 (ATCC 33152, Eurofins, France) was used as bacterial model for the method development. It was cultivated on a GVPC agar plate (Glycine Vancomycin Polymyxin Cycloheximide) (Biomerieux, France) at 37 °C. For the titration of bacteria suspensions, 100 µL were sampled and resuspended in 1 mL of sterile water before being placed at 95 °C for 10 min. From this lysis, 5 μL were used in order to perform a direct quantitation via qPCR.

For validation, a stock solution of DNA of *Legionella pneumophila* was used. The DNA was extracted from a *Legionella pneumophila* culture while using the phenol-chloroform method, as previously described [21]. DNA solutions were titrated via qPCR using an IQ-Check *L. pneumophila* kit (Biorad, France). 

### 2.3. Samples

#### 2.3.1. *Legionella pneumophila* Bacterial Suspensions

A suspension of bacteria was realized from a pure culture on a GVPC agar plate culture media in sterile water and then adjusted to 0.6 UDO. mL^−1^ at 600 nm. The solution was diluted (by serial dilutions) in order to obtain 100 mL of five solutions that were diluted to factors of 5.10^4^, 1.10^5^, 5.10^5^, 1.10^6^ and 1.10^7^. An initial titration of each obtained dilution was realized. To this aim, 100 µL of each dilution was sampled and resuspended in 1 mL of sterile water before being placed at 95 °C for 10 min. Further, 5 µL were used in order to quantitate the sample using qPCR.

#### 2.3.2. Raw Water from Industrial Cooling Tower 

The water samples were obtained from 24 cooling systems from March to April 2015. The samples were collected in 1 L plastic sterile bottles (20 mg/L sodium thiosulfate, VWR) and stored at 4 °C until analysis in accordance with the French normative procedure XPT 90-471. The samples were analyzed with this newly developed method and the results were compared with ones that were obtained by an independent COFRAC laboratory. In the two methods, 100 mL of each sample were filtered through a 0.45 μm polycarbonate filter (Whatman^®^ Nuclepore Track-Etched Membranes, Sigma–Aldrich, St. Louis, MO, USA) in a parabolic polypropylene funnel (516-7024, VWR). The detection of *Legionella* that was conducted by the COFRAC laboratory was performed while using a standardized PCR method (Aquadien kit for extraction (Biorad, France) and IQ-Check *L. pneumophila* kit (Biorad, France)). 

For the protocol that was developed in this paper, the filter was transferred into a tube containing 1.5 mL of a lysis buffer (the lysis buffer contained TE and chelex resin (Biorad, France)), and it was heated for 15 min. at 95 °C before DNA extraction. After 5 min. of decanting the chelex resin, 1 mL of supernatant was recovered using the DNA purification procedure described.

#### 2.3.3. Food Sample

Different types of beverages were selected for analysis: wine, soda, milk, pineapple juice, orange juice, strawberry juice, and apple juice. For the non-filterable matrices, beverages included milk, soda with pulp, grapefruit juice, strawberry juice, and orange juice. Next, 10 mL was centrifuged for 30 min. at 4000 rpm. The pellet was resuspended in a 1.5 mL TE buffer before adding the lysis buffer. For the other matrix, 100 mL was filtered in the same conditions as the raw water.

For wine, milk, and orange juice, two different commercial products were used. The experiments were performed (n = 4) when one commercial product was used and (n = 2) when two commercial products were used. The results were the average of the four independent experiments.

#### 2.3.4. Medical Sample

Extraction was performed on blood, stools, and urine. Blood was provided by the Établissement Français du Sang. Stools and urine were provided from patients that provided written informed consent to participate to this study in accordance with the Declaration of Helsinki as revised in 2008. The extraction was performed on 500 µL of blood, 1 mL of urine, and 100 mg of feces.

Extracting the liquid sample was realized after adding the lysis buffer, followed by a thermal lysis at 95 °C for 10 min.

For the feces, the cell lysis was realized after adding the lysis buffer and glass beads. It was then vortexed for 5 min. This lysis was followed by a treatment with proteinase K during 15 min. before its inactivation at 95 °C for 5 min. 

The experiments were realized in triplicate from each sample.

### 2.4. Procedure of DNA Purification

After cell lysis, 100 µL of a PLL solution at 10 mg/mL was added and mixed by inverting. The sample was then transferred into a new tube containing 80 mg of a weak cationic exchange resin (10 meq/g) and then mixed slowly by inversion. After 5 s of centrifugation at 10,000× *g*, the supernatant was discarded. The elution was performed after adding 100 μL of a TE buffer pH 8, followed by agitation (1500 rpm for 10 min.) at 75 °C while using a thermomixer instrument (Bioshake iQ, Q.Instruments). The elution fraction was recovered and stored at −20 °C for further analysis. 

### 2.5. qPCR

Next, 5 mL of the sample that was obtained either via direct lysis or after the purification procedure was used as a template for qPCR. The kit used for quantification was the iQ-Check^®^ Quanti *L. pneumophila* Real-Time PCR (Biorad, France). The mix for PCR was realized by following the manufacturer’s instructions. The qPCR device that was used in this study was the ChromoV device 20 (Biorad, France). 

Quantification was realized using the four standards (called Qs) that were supplied with the PCR kit that allowed us to realize a standard curve. Moreover, this kit allowed for us to assess the inhibitory potential of a sample, thanks to a synthetic DNA included in each amplification mixture. This synthetic DNA exhibited 5′ and 3′ ends, which were similar to the target and amplified by the same primers and those used for *L. pneumophila.* When compared to *L. pneumophila* PCR amplicon, synthetic DNA presented a modified sequence detected while using a specific probe with a different reporter (HEX fluorophore). A sample was inhibited if the Ct of the internal control (HEX channels) measured in a sample was superior to the average Ct of the internal control ± 3-fold the standard deviation of Ct values. The average Ct of the internal control was measured in the four standards (QS). 

## 3. Results

### 3.1. Mechanical Mechanism of Elution

The originality of our process was based on the use of a mechanical force in order to elute DNA from the resin-PLL complex. First, the agitation force necessary for elution was determined. As such, 1 mL of sterile water was spiked with about 19,000 GU (Genome Unit) of genomic *legionella* DNA. Subsequently, DNA was purified according to the DNA purification procedure, as described in the experimental section. During this step, six agitation speeds were tested: 0, 500, 1000, 1500, 2000, and 2500 rpm. 

A qPCR was performed before and after the process to assess the percentage recovery, as follows:

$$\%\;recovery\;=\;\frac{GU\;amount\;measured\;after\;purification}{initial\;GU\;amount}$$

**Figure 2:**
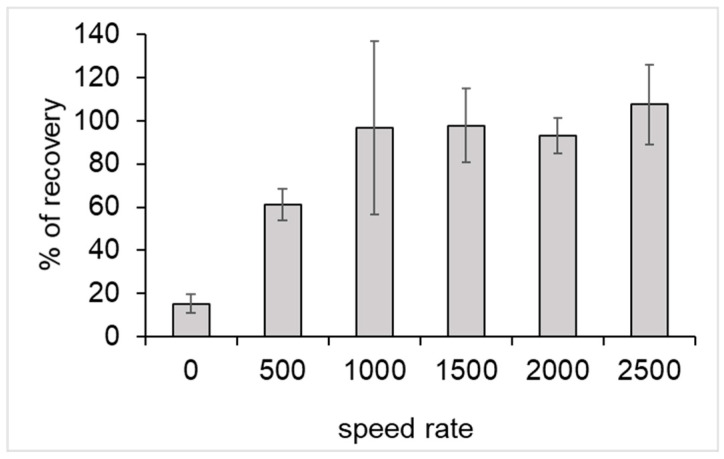
Percentage recovery of DNA in the speed rate agitation function. Error bars represent the standard deviation for n = 3.

A speed rate of at least 1000 rpm was necessary for obtaining a total recovery of initial DNA (Figure 2).

For the following experiments, the speed rate of elution was fixed at 1500 rpm. At this speed, it was verified that only DNA was eluted and no PLL was found in the elution buffer (data not shown).

### 3.2. Impact of DNA Concentration on Process Efficiency 

The method’s linearity was assessed based on bacteria solutions in order to evaluate the potential interferences that are brought about by residues (cellular debris) of bacteria lyses and purification. Five titrated bacterial suspensions, concentrated at 2951, 4571, 33,884, 45,709, and 457,088 GU/mL, were used to spike 100 mL of sterile water. After thermal lysis, DNA was purified via the described process.

**Figure 3:**
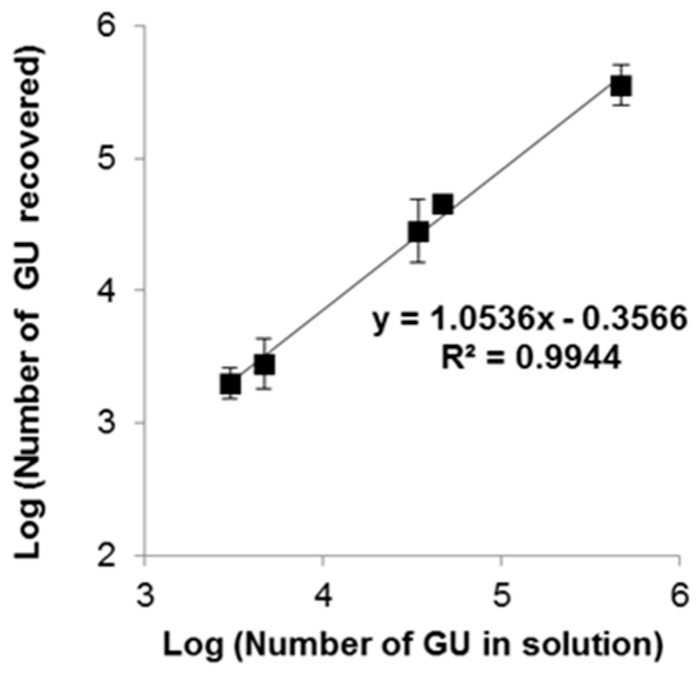
Assessment of the method’s linearity. Curves represent the log of the expected number of GU in the solution as a function of the number of GU recovered after extraction. Error bars represent the standard deviation for n = 3.

The percentage recovery ranged from 66% to 98%. Data showed strong linearity, with a slope and Y intercept of 1.05 and −0.35, respectively, with a good correlation coefficient (R^2^ = 0.99) (Figure 3).

### 3.3. Impact of pH on Process Efficiency 

Although pH could be an important parameter in our process, we found that ionic interactions and the interaction between PLL and the weak cation exchange resin cause the DNA-PLL interaction. PLL had a pKa near 10. Interaction with resin was described by the manufacturer as optimal for a pH range between 5 and 0.5–1.5 pH units greater than the pI of the PLL. Therefore, the theoretically optimum pH for the DNA capture was between 5 and 8.5.

**Figure 4:**
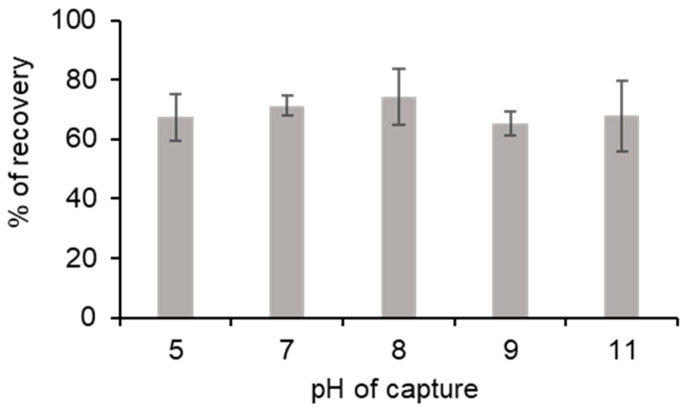
Influence of the pH on DNA capture. Five pH capture samples were tested. The percentage recovery corresponded to the percentage of DNA recovered after the process ended. Error bars represent the standard deviation for n = 3.

The process efficiency was tested with different pH levels in the capture solution. Indeed, 132,770 GU of *Legionella pneumophila* were incorporated in a 1 mL Tris-HCL-EDTA buffer, with a pH adjusted between 5 and 11. The impact of the solution’s pH was assessed by calculating the percentage recovery after the process ended. Whatever the pH of the capture solution was, the percentage recovery at the end of the process was between 65.3% and 74.4% (Figure 4). ANOVA was performed on the results and there was no significant difference between pH conditions (F 4,10 = 0.801, ns). The fact that pH did not impact DNA capture allowed for us to engage in the purification process without correcting the pH sample.

### 3.4. Assessment of the Purification Process on Real Samples

#### 3.4.1. Cooling Tower Systems

In total, 24 different waters from various cooling tower systems were collected. The cooling towers were selected because, in routine analyses, the DNA that was obtained from extraction often contained inhibitors and it was necessary to dilute the sample for their analyses. All of the samples were extracted while using the standardized method, which required a dilution factor between 5 and 50 to be analyzed (details are provided in Supplementary Materials Table S1). In comparison, 23 out of 24 samples that were extracted with our method could be analyzed directly without dilution. In total, 10 out of 24 samples were diagnosed a negative by the COFRAC laboratory, and they were used to access the new method’s extraction efficiency. The samples were divided in two equal, 100 mL parts: one part was directly analyzed, while the other was spiked with an amount of *L. pneumophila* that was equivalent to 135,000 GU.

**Figure 5:**
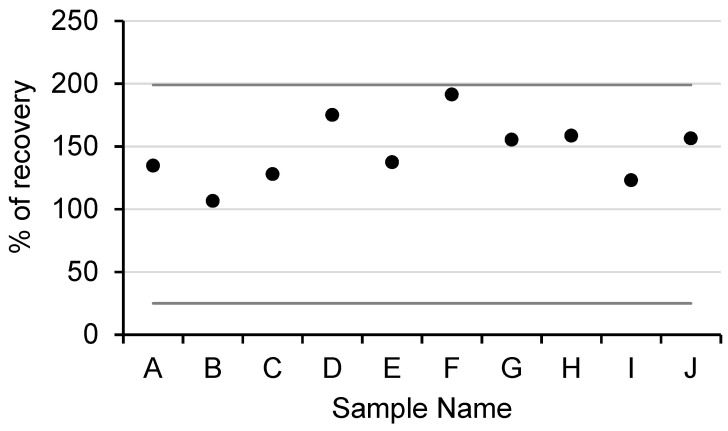
Assessment of the purification process on real inhibited samples. DNA evaluation recovery of 10 negative samples spiked with 135,000 GU of *L. pneumophila*. Dark gray lines represent the accepted recovery yields described in the French normative association XPT 90-471.

In the spiked samples, the percentages of genomic DNA recovery ranged between 106% and 191% (Figure 5). The level superior to 100% could be explained by the well’s PCR efficiency variations (i.e., either in initial quantitation of the spiked or in the quantitation after extraction). Indeed, a difference of the 10% PCR efficiency in the well led to a difference (factor 2) in the quantification. The obtained percentages followed the French normative association requirements (XPT 90-471, April 2006), which demanded recovery yields between 25% and 199% (line in dark gray on the graphic), which validated the process. 

#### 3.4.2. Beverage Samples

Samples of commercial beverages that were known to pose DNA extraction problems were selected on the basis of their composition for their high content of polysaccharides, polyphenols, or proteins, all of which are strong PCR inhibitors. All of the products were commercial products that were artificially doped with 1000 GU of genomic DNA from *Legionella pneumophilla*. The choice was made to keep this bacterium for spiking, because it was not naturally present in samples or a source of classical contamination. The extraction yield was directly carried out. No PCR inhibition was found after extraction.

**Figure 6:**
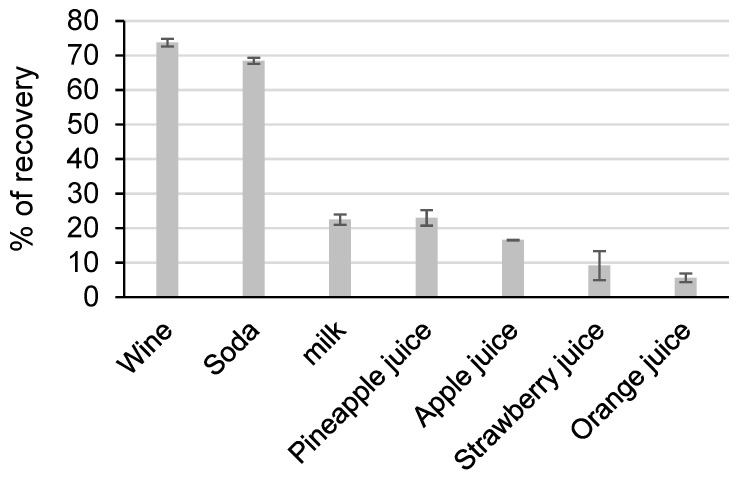
Percentage of DNA recovery from different beverage matrices. Standard deviation was calculated from four independent experiments.

The extraction yields ranged from 5.6% to 73.8% and varied greatly depending on the matrix (Figure 6).

Good extraction yields were obtained for wine and soda, but low percentage recovery values were found for other matrices, i.e., milk and juices.

#### 3.4.3. Medical Samples

Medical samples contained PCR inhibitors, such as bile salts in feces, heme in blood, and urea in urine.

The following medical samples were used: urine, blood, and feces artificially spiked with genomic DNA from *Legionella pneumophila* 57,258 GU.

The extraction yields were 70.6% ± 8.6, 71% ± 8.4, and 108.1% ± 8.1 for feces, blood, and urine, respectively. Whatever the medical matrix, a high level of DNA extraction was observed without PCR inhibition. 

## 4. Discussion

Obtaining a sufficient amount of DNA without inhibitors is a challenge in a number of molecular biology fields and applications. The procedure of DNA extraction is usually optimized, either for the target or the origin of the samples [22,23,24,25,26]. Moreover, the issues concerning extraction are the same, which leads to the extraction of a sufficient amount of DNA and a sufficient good quality to avoid inhibitors. DNA extraction kit performances were not assessed, as we assessed specific samples in this study.

When we developed this homemade process for DNA extraction, our aim was to respond to a specific problem: the presence of PCR inhibitors in a water environmental sample, which made molecular biology studies difficult to perform. Through the example of *Legionella* contamination in a cooling tower, we assessed a DNA extraction and purification process. The process efficiency was assessed while using the French normative document XPT 90-471. The absence of inhibition on 24/25 samples was inhibited by a standard analysis was an interesting step for assessing the extraction process for different types of matrices. Whatever the matrix, this method considered the extraction protocol to be suitable for removing inhibitors with little adaptation being required for different samples. 

We observed a large variability of matrix yields: 70.6% to 108.1% for the biological matrix and 5.6% to 73.8% for the beverage matrix If it is easy to conclude that the heme, urea, and bile salt do not provide major problems for the PLL to complex DNA, it becomes more complicated to explain the difference of results that were obtained between wine and juice. One hypothesis could be that the evolution of the composition in polyphenol during wine fermentation [27] could explain better yields that were obtained in this matrix. Concerning the milk matrix, the presence of lipids could limit the interaction of the DNA-PLL polyplex with the resin.

The process was efficient when capturing DNA and removing it without inhibitors, but, in those tests, some molecules limited the capture of DNA by the PLL. 

For future work, it will be interesting to assess this method during microbiota analysis. Indeed, the DNA extraction method is crucial for microbiome analysis, and several studies have highlighted the importance of the extraction methods on the obtained results [22,28].

## 5. Patents

Cadiere A., Faye C., Francois C., Garrelly L.: Method for purifying and concentrating nucleic acids. WO 2017005754 A1.

## Supplementary Materials

The following are available online at https://www.mdpi.com/2076-2607/8/12/1901/s1, Table S1: Dilution factor required to remove inhibition with the standard protocol.
