# Supplementary material for: The Utilization of Linear Polylysine Coupled with Mechanic Forces to Extract Microbial DNA from Different Matrices
Source: Microorganisms. 2020 Nov 30;8(12):1901. doi: 10.3390/microorganisms8121901 (PMC7760326; doi:10.3390/microorganisms8121901)
Supplement: Supplementary file 1 [file microorganisms-08-01901-s001.pdf]

microorganisms

Supplementary Materials

Utilization of linear polylysine coupled with mechanic forces to extract microbial DNA from different matrix

Celia François[1], Celia Martinez[1], Clement Faye[1], Nathalie Pansu[2,3], Catherine Dunyach-Remy[2,3], Laurent Garrelly[1], Benoit Roig[4] and Axelle Cadiere[4,*]

Supplementary Table S1. Dilution factor required to remove inhibition with the standard protocol

Sample	Dilution factors applied for PCR analysis to remove inhibition with the standard protocol
1	5
2	10
3	10
4	10
5	10
6	5
7	50
8	10
9	10
10	50
11	10
12	50
13	5
14	10
15	10
16	10
17	50
18	10
19	5
20	10
21	5
22	10
23	5
24	5
